# Role of microRNA alternation in the pathogenesis of gouty arthritis

**DOI:** 10.3389/fendo.2022.967769

**Published:** 2022-08-11

**Authors:** Zhipan Luo, Fan Yang, Shaocheng Hong, Jianpeng Wang, Bangjie Chen, Liangyun Li, Junfa Yang, Yan Yao, Chenchen Yang, Ying Hu, Shuxian Wang, Tao Xu, Jun Wu

**Affiliations:** ^1^ The First Affifiliated Hospital, Anhui Medical University, Hefei, China; ^2^ Inflammation and Immune Mediated Diseases Laboratory of Anhui Province, Hefei, China; ^3^ Anhui Institute of Innovative Drugs, Hefei, China; ^4^ School of Pharmacy, Anhui Medical University, Hefei, China; ^5^ Institute of clinical pharmacology, Anhui Medical University, Hefei, China; ^6^ Geriatric Department, The First Affifiliated Hospital of USTC, Division of Life Sciences and Medicine, University of Science and Technology of China, Hefei, China

**Keywords:** MicroRNA, gouty arthritis, hyperuricemia, cellular signaling pathway, biomarker

## Abstract

Gouty arthritis is a common inflammatory disease. The condition is triggered by a disorder of uric acid metabolism, which causes urate deposition and gout flares. MicroRNAs are a class of conserved small non-coding RNAs that bind to the 3’ untranslated region (UTR) of mRNA and regulate the expression of a variety of proteins at the post-transcriptional level. In recent years, attention has been focused on the role of miRNAs in various inflammatory diseases, including gouty arthritis. It is thought that miRNAs may regulate immune function and inflammatory responses, thereby influencing the onset and progression of the disease. This article mainly reviewed the roles of miRNAs in the pathogenesis of gouty arthritis and prospected their potential as diagnostic and prognostic relevant biomarkers and as possible therapeutic targets.

## 1 Introduction

Gouty arthritis (GA) is an inflammatory joint disease with a prevalence of 3.9% of all adults in the United States, 5.2% for men, and 2.7% for women (1). As a disorder of uric acid metabolism, this disease is mainly caused by the deposition of monosodium urate crystals (MSU) in the joint capsule, bursa, bone, and cartilage, ultimately causing joint damage and even deformity (2). With gout flares, the pain increases and seriously affects the patient’s quality of life. In addition, gout is closely associated with metabolic comorbidities that can lead to myocardial infarction, type 2 diabetes, chronic kidney disease, and premature death (3, 4). The treatment of gouty arthritis attacks is mainly to control pain and suppress joint inflammation, such as the use of non-steroidal anti-inflammatory drugs, glucocorticoids, etc. The long-term management of patients with gouty arthritis focuses on uric acid-lowering therapy to reverse hyperuricemia and thus prevent gout attacks (5, 6). Despite new treatment strategies and a good understanding of the pathogenesis of gouty arthritis, recurrent attacks continue to occur after treatment (7).

MiRNA is a conserved short-stranded non-coding RNA of approximately 23nt in length, and they were first identified in Caenorhabditis elegans in 1993 (8). miRNAs are first transcribed in the nucleus as longer primary miRNAs (primary miRNA, pri-miRNA), then processed in the nucleus by Drosha into hairpin RNAs of 60-70 nucleotides, i.e. precursor miRNAs (miRNA precusor, pre-miRNA), which are transported out of the nucleus with the help of the Exprotin-5 complex and sheared in the cytoplasm by Dicer to become mature miRNAs (9, 10).Current studies have shown that miRNAs are highly conserved evolutionarily (11). A miRNA can regulate the activity or stability of multiple target genes by recognizing and inducing the assembly of the RNA silencing complex (RISC) with the miRNA response element (MRE) in the mRNA 3’ untranslated region (UTR) region of the target gene, and multiple microRNAs can also synergistically regulate the same target gene (12, 13). More than 1,000 miRNAs have been identified in human cells, while more than 500 microRNAs in the human body (14). Although the functions of miRNAs are not fully understood, relevant studies have shown that miRNAs are involved in various processes, including cell differentiation, metabolism, and inflammation (15).

Recent research has demonstrated that miRNA plays an essential role in the pathogenesis of common nonautoimmune inflammatory diseases, including gouty arthritis (16). Although some studies have attempted to elucidate the crucial role of miRNAs in the pathogenesis of gouty arthritis, their analyses have always been conducted in a single direction. They have not diversified to integrate multiple fields of study. Therefore, this paper reviews the various regulatory mechanisms of miRNAs in developing gout, including its relationship with uric acid metabolism, classical inflammatory signaling pathways, and bone erosion. On this foundation, we considered the promise of miRNA as a potential diagnostic and prognostic marker for gout and as a therapeutic target.

## 2 Overview of gouty arthritis

**Figure 1:**
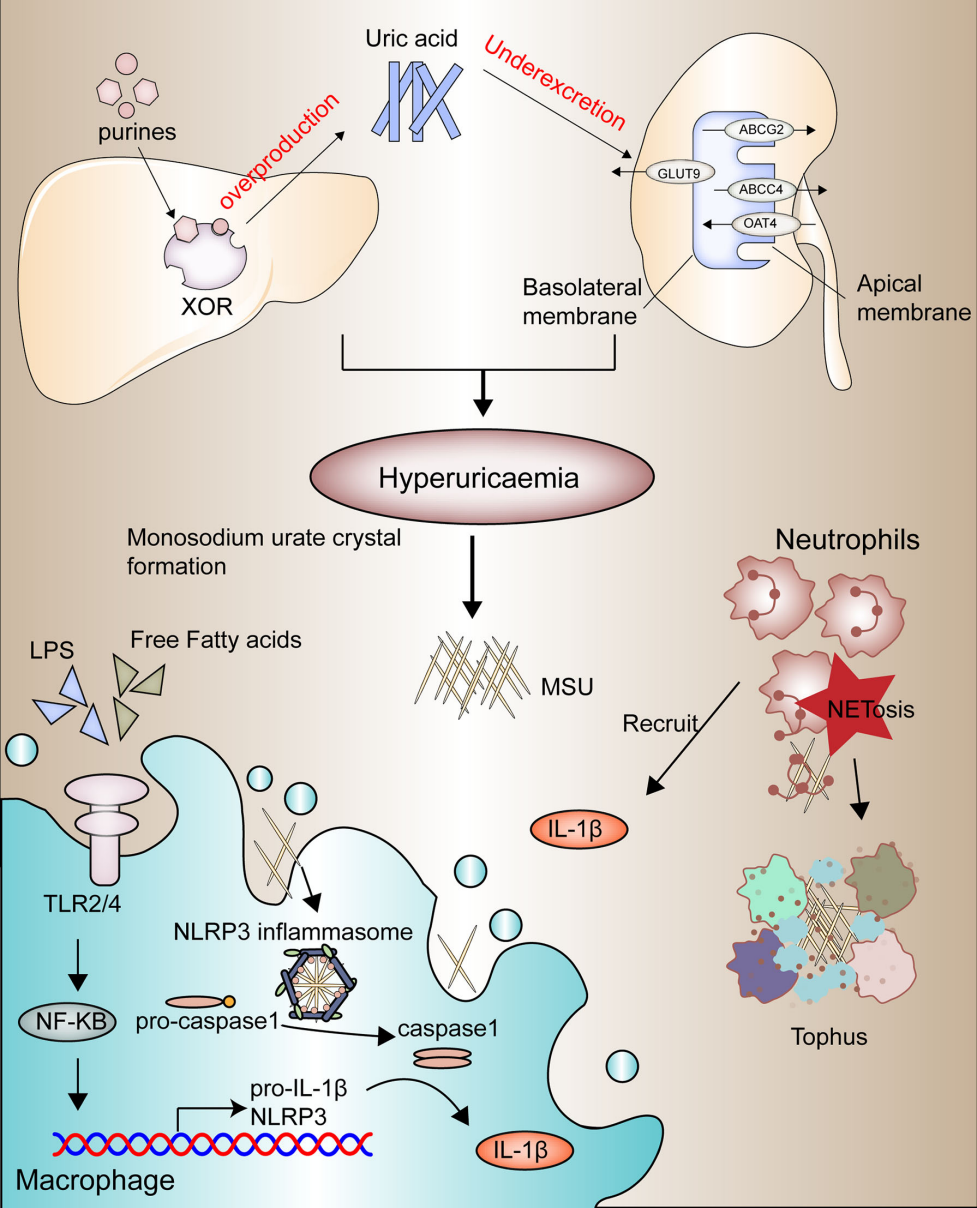
Four stages in the pathophysiological development of gouty arthritis.

Gouty arthritis (GA) is characterized by swelling and heat pain in one side of the joint, causing joint dysfunction, deformity, and even disability (17). Epidemiology reports the current range of gout incidence at 0.6-2.8 per 1000 people per year. The prevalence of gout has continued to increase worldwide in recent decades, probably due to the aging of society’s population and changes in dietary patterns (18–20). The development of gout is based on four pathophysiological stages, the first two of which are hyperuricemia and the formation and deposition of sodium urate crystals, the leading causes of which are disorders of purine metabolism and dysregulation uric acid secretion (**Figure 1**). The latter two components are mainly gout flares triggered by acute inflammatory reactions and irreversible bone erosion caused by the deposition of tophi in advanced gout (21).

Usually, the intake of purines is converted by xanthine oxidase in the liver to uric acid, which is excreted mainly through the kidneys (22). The renal tubular transporters such as OAT4 and GLUT9 are responsible for the reabsorption of uric acid (23, 24), while ABCG2 and ABCC4 are responsible for the secretion of uric acid (25, 26). And once a high purine diet such as alcohol, seafood, and meat is accompanied by impaired renal excretion function, it will lead to hyperuricemia. Once a high purine diet is accompanied by inadequate excretion by the kidneys, it can lead to hyperuricemia (27, 28).

Due to the high concentration of urate, along with other physical and chemical environments, MSU is generated and deposited in the joint cavity, which in turn acts as foreign bodies to recruit neutrophils and macrophages and initiate toll-like receptor (TLR) and NLRP3 inflammasome signaling pathways to activate innate immunity (29–31). Current studies suggest that the acute flares of gout depend on two switches, one of which is the activation of the TLR2/4-NF-κB signaling pathway within macrophages or monocytes, which promotes the synthesis of pro-IL1-β and significant components of the inflammasome, and that this activation is associated with the influence of large amounts of free fatty acids, intestinal flora or other microorganisms (32, 33). Stimulating sodium urate crystals is another critical point of activation of gout inflammation, which activates the NLRP3 inflammatory pathway by promoting the assembly of inflammatory vesicles, thereby promoting the conversion of pro-caspase-1 to caspase-1 and the release of large amounts of pro-inflammatory factors such as IL-1β (34–36).

These pro-inflammatory factors recruit more neutrophils to exacerbate the inflammatory response; however, along with the inflammatory death of large numbers of neutrophils, activated neutrophils release extracellularly depolymerized chromatin and intracellular granule proteins, called neutrophil extracellular traps (NETs), to trap and break down inflammatory factors to relieve gout flares (37, 38). This chronic inflammatory response develops into the advanced disease characterized by tophi (**Figure 1**), a microenvironment of adaptive and innate immune cells, MSU, and fibroblasts, promoting bone resorption by osteoclasts and reducing bone formation osteoblasts, and ultimately causes bone erosion (39, 40).

## 3 The function and mechanism of miRNAs in GA

Extensive studies illustrate that abnormal expression in miRNAs occurs during the pathophysiology of gouty arthritis (15, 41, 42). Only 10% of the population with high uric acid has positive signs of gouty arthritis, which may be associated with different genetic backgrounds, and miRNA sequence alterations affect the genetic susceptibility background (43). In addition to this, human genome-wide association studies (GWAS) have identified many loci associated with hyperuricemia and gout, and these single nucleotide polymorphism (SNP) loci are mainly associated with the coding of uric acid transporter genes (44, 45). Further studies have illustrated the ability of miRNAs to regulate inflammatory immune-related processes in gouty arthritis (46, 47).

Over the past years, attempts have been made to identify aberrantly expressed MiRNAs in gout to explore the role of these molecules in the pathogenesis of gout. In 2014 Tae-Jong Kim et al. first investigated the role of Mir-155 in acute gouty arthritis (48). So far, studies on gouty arthritis have been divided into omics-based high-throughput studies and *in vitro* cellular models stimulated with MSU or *in vivo* animal models. A large number of meaningful results have now been identified. Therefore, we will summarize the relationship between miRNAs and gout pathogenesis in terms of hyperuricemia, inflammatory immunity, and bone erosion and look at their diagnostic and therapeutic value based on the last five years of publication and older but more classic literature

### 3.1 Involvement of miRNAs in the hyperuricemia

Hyperuricemia (HUA) is the prodromal stage of gout attack and a common clinical feature in the course of gouty arthritis. The inability to excrete uric acid from purines promptly leads to a series of disturbances in the metabolic environment and can even cause damage to liver and kidney function (49). As the body’s primary urate handling organ, the kidney generally relies on renal tubular urate transport proteins, such as URAT1, GLUT9, and ABCG2, to regulate uric acid excretion (26, 44). It was reported that C421A polymorphism enhanced the degradation of ABCG2 in a miRNA-dependent manner and that the use of inhibitors of miR-519c and miR-328 reversed this translational repression (50). Sun, W. et al. reported that Xie-Zhuo-Chu-Bi-Fang could upregulate miR-34a and downregulate URAT1 to treat hyperuricemia (51). In addition, miR-143-3p can directly target the 3’UTR of GLUT9 in renal tubular epithelial cells to reduce uric acid reabsorption and inflammatory response (52). In a clinical study, miR-155 was elevated in the serum of HUA patients, and subjects with urate deposition had higher miR-155 than those without deposition findings (53). *In vitro* experiments also revealed that miR-155 was elevated in high-uric acid-stimulated venous endothelial cells (HUVEC) and inhibited eNOS expression causing endothelial cell dysfunction (54).

Similarly, hyperuricemic stimulation led to the downregulation of miR-92a, thereby inhibiting vascular neogenesis through the KLF2-VEGFA axis (55). Hong Q et al. also found that miR-663 could act on the transcript of TGF-β1 to regulate PTEN to inhibit endothelial cell migration (56). These studies also suggest that high uric acid causes cardiovascular damage and explains the correlation between gout and cardiovascular disease such as myocardial infarction. And miRNAs also play a precise regulatory role in liver and kidney function damage caused by excessive uric acid. Uric acid damages renal interstitial fibroblasts by downregulating miR-9 and causing activation of NF-KB and JAK-STAT pathways (57). Besides, Chen, S et al. also reported that overexpression of miR149-5p inhibited FGF21 expression and attenuated uric acid-induced lipid deposition in hepatocytes (58). Chi, K et al. found that HOTAIR competitively binds miR-22 in hyperuricemia to regulate NLRP3 inflammasome activation to promote endothelial cell pyroptosis and exacerbate renal injury (59). Recent studies have also reported a decrease in miR-30b and an increase in IL-6R in serum urine and kidney tissue in a mouse model of HUA (60). The above study we summarized in the **Table 1**. In summary, miRNAs are involved in the development of hyperuricemia and play an important role, and targeting miRNA processing may provide new insights for the future treatment of hyperuricemia.

**Table 1:** The miRNAs involved in Hyperuricemia.

miRNA	Result	model	reference
miR-22-3p↓	NLRP3 inflammasome↑, pyroptosis↑	*In vivo* and *in vitro*	(59)
miR-30b↓	IL-6R↑	*In vivo*	(60)
miR-34a↑	URAT1↓	*in vivo*	(51)
miR-149-5p↑	FGF21↓, hepatocytes lipid accumulation↑	*In vitro*	(58)
miR-143-3p↓	GLUT9↑	*In vitro*	(52)
miR-9↑	NF-KB↓, JAK-STAT↓, injury in NRK-49F↓	*In vitro*	(57)
miR-92a↓	KLF2↑, VEGFA ↓, Angiogenesis↓	*In vitro*	(55)
miR-663↑	TGF-β1↓, PTEN↓, migration↓	*In vitro*	(56)
miR-155↑	eNOS↓, endothelial dysfunction	*In vitro*	(54)

a “↑” indicates elevated expression or facilitation.

b “↓” indicates reduced expression or inhibitory effect.

### 3.2 Role of miRNAs in the regulation of immune-inflammatory responses

#### 3.2.1 miRNAs and TLR2/4/MyD88/NF-KB pathway in GA

**Figure 2:**
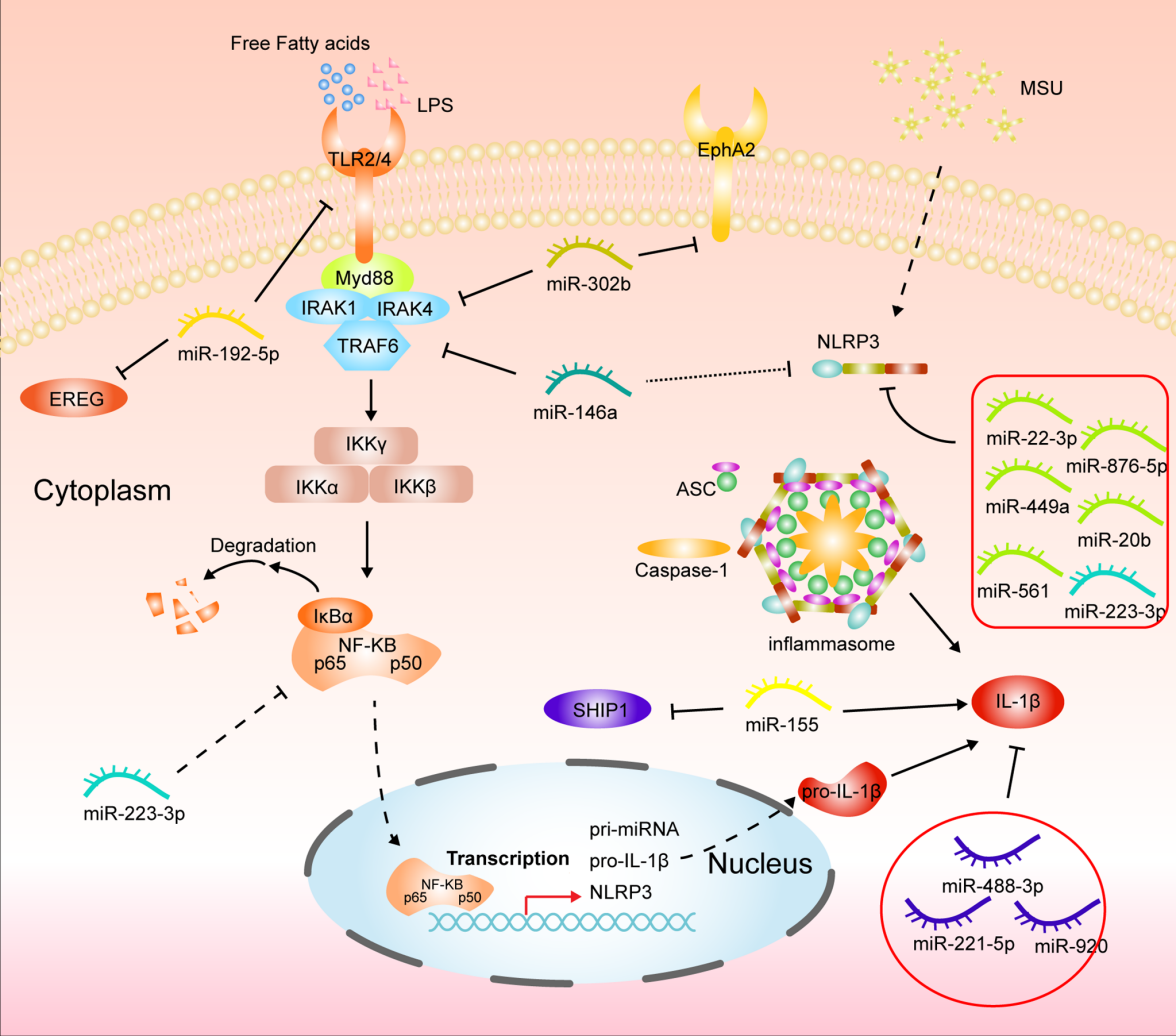
Mechanisms of miRNAs in gouty arthritis regulating inflammatory pathways in monocytes/macrophages.

The onset of gouty arthritis results from an inflammatory immune response triggered by MSU deposition. Two pathways mediate the main molecular mechanisms: activation of the TLR-related NF/KB signaling pathway and activation of the inflammatory vesicle NLRP3, respectively. The former is mainly microbial or free fatty acids activating Toll-like pattern recognition receptors, mainly TLR2/4, recognizing the downstream signaling molecule myeloid differentiation factor 88 (MyD88) for intracellular signaling and finally leading to the activation of NF-KB (31, 35, 61). Numerous studies have illustrated the significant correlation between miR-192 and NF-KB pathway. For example, miR-192-5p effectively alleviated tumor progression by inhibiting the IRAK1/NF-κB pathway in endometrial cancer (62, 63). Recently, Lian, C et al. found that miR-192-5p in MSU-treated synovial fluid mononuclear cells (SMFCs) and THP-1 could target TLR4 to inhibit NF-KB pathway activation reducing TNF-a and IL-1β release (**Figure 2**) (64). MiR-146a is the first regulator that is involved in innate immunity. It has been reported that miR-146a can regulate key downstream adaptor molecules of TLR in sepsis by complementary pairing with the 3’UTR base sequence of TNF receptor-associated factor 6 (TRAF6) and IL-1 receptor-associated kinase 1 (IRAK1) genes, thereby inhibiting the activity of TLR signaling pathway and thus inhibiting NF- κB signaling pathway to exert inflammatory suppressive effects (65). Another study indicates miR-146a alleviates inflammation in acute gouty arthritis in rats *via* TLR4/MyD88/NF-KB signaling pathway. And further study demonstrated that miR-146a knockout mice promoted the development of gouty arthritis by upregulating TRAF6 and IRAK-1 expression compared to wild type (**Figure 2**) (66, 67). Ma, T et al. illustrated that MicroRNA-302b could directly bind to the 3’ UTR of IRAK4 and EphA2 in an *in vivo* and *in vitro* model of gout to inhibit activation of the NF-KB pathway to reduce IL-1β (**Figure 2**) (68). Therefore, targeting the TLR-mediated NF-KB pathway *via* miRNA can be a promising approach for GA treatment.

#### 3.2.2 miRNAs and NLRP3 inflammasome pathway in GA

NLRP3 is the most comprehensive subfamily studied in the nucleotide-binding oligomerization domain-like receptor (69). NLRP3 inflammasome comprises NLRP3, recruitment domain, an adaptor protein, apoptosis-associated spot like protein (ASC), and caspase-1, expressed in many immune cells. In gouty arthritis, MSU crystal acts as a ligand to bind and activate NLRP3 inflammasome. After the conformational change of NLRP3 protein, it polymerizes with ATP to form a protein oligomer. It then recruits pro-caspase-1 and ASC through its effector domain PYD, The caspase-1 precursor was automatically catalyzed into the active form, and proIL-1β was enzymolysis into IL-1β (70, 71). Current studies have confirmed that miRNAs are critical regulators of the NLRP3 inflammasome pathway (72). In recent years, it has been reported that circHIPK3 can act as a molecular sponge to adsorb miR-561 to competitively bind NLRP3 mRNA to reduce inflammation in GA (64). Upregulation of miR-20b expression and downregulation of NLRP3 protein was also found in macrophages with HOTAIR knockdown (73). Wang, X et al. reported that miR-223-3p and miR-22-3p could reduce inflammatory effects in monocytes and mouse models of gout by interacting with the 3’ untranslated region segment of NLRP3 mRNA (74). And Tian, J et al. also found that miR-223 could target NLRP3 mRNA in MSU-induced rat models and fibroblast-like synoviocytes to inhibit inflammation and cellular pyroptosis. Further studies confirmed that miR-223 deficiency exacerbated the swelling index of MSU-induced joint inflammation and intensified inflammatory cell infiltration and cytokine release, including IL-1β and MCP-1, compared to WT mice (75, 76). Similarly, the expression of NLRP3 was dramatically upregulated in Bone marrow-derived macrophages (BMDMs) from miR-146a KO mice (67). Besides, the active ingredients of some Chinese traditional medicines also exert anti-inflammatory effects in GA by regulating the miRNA/NLRP3 axis. Total glucosides of paeony alleviate *in vitro* MSU-induced inflammation in macrophage THP-1 *via* the MALAT1/miR-876-5p/NLRP3 cascade pathway. Wang Y et al. also reported that Tripterine alleviates GA by modulating miR-449a to act directly on NLRP3 mRNA and inhibit its expression (**Figure 2** **) (** 77, 78).

#### 3.2.3 miRNAs and other mechanisms of inflammation in GA

In GA, when inflammatory pathways in macrophages are activated, they are polarized toward the M1 phenotype and release large amounts of pro-inflammatory factors such as TNF-a, IL-1β, and MCP-1. miRNAs also play a regulatory role in this process. MiR-449a and miR-192-5p can target NLRP3 and epiregulin (EREG) to inhibit macrophage M1 polarization in gout (77, 79). Apart from this, miRNA-488-3p and -920 both can interact with the 3’UTR of IL1-β to exert anti-inflammatory effects (80). Li, G. et al. also reported that miR-221-5p represses IL1-β expression in acute gouty arthritis to regulate the inflammatory response (81). Although Jin, H. M et al. reported that overexpression of miR-155 *in vitro* reduced SHIP-1 levels and promoted MSU-induced TNF-a and IL-1β production (**Figure 2**), it was later indicated that there was no remission of GA in miR-155 knockout mice compared to wild type (48, 82). Inflammatory factors tend to infiltrating immune cells, such as neutrophils, which on the one hand, worsen inflammation.

On the other hand, large numbers of neutrophils accumulate inflammatory death, chromatin remodeling, and ejection outside the cells to form NETs, called NETosis. These aggregated NETs can trap pro-inflammatory factors and act in conjunction with some anti-inflammatory factors to reduce the development of inflammation. Yet, few studies on the relationship between miRNAs and NETs in gout. Recently, it has been shown that MSU stimulation can significantly increase miR-3146 expression in neutrophils, accompanied by the formation of many NETs. In contrast, treating rats with antagomir-3146 reduced NETs formation and relief of joint swelling and inflammation, suggesting that early NETs formation exacerbates GA and that miR-3146 plays a role vital role before NETs formation (83). However, the current research on NETs is still inadequate, and how miRNAs regulate the development of NETs in gout is still unclear. Further studies are needed to reveal the potential mechanisms to help people understand gout and identify potential therapeutic targets. We conclude with a summary of miRNAs involved in inflammatory immunity. (**Table 2**)

**Table 2:** The miRNAs involved in inflammatory immunity.

miRNA	target gene/pathway	Role in GA	model	reference
miR-223-3p	NLRP3/NF-KB	–	*In vivo* and *in vitro*	(74–76)
miR-449a	NLRP3	–	*In vivo* and *in vitro*	(77)
miR-22-3p	NLRP3	–	*In vivo* and *in vitro*	(74)
miR-3146	SIRT1	+	*In vivo* and *in vitro*	(83)
miR-876-5p	NLRP3	–	*In vitro*	(78)
miR-20b	NLRP3	–	*In vitro*	(73)
miR-561	NLRP3	–	*In vitro*	(64)
miR-192-5p	TLR4 EREG	–	*In vivo* and *in vitro*	(64, 79)
miR-221-5p	IL1-β	–	*In vitro*	(81)
miR-146a	TLR4/MyD88/NF-KB TRAF6/IRAK1/NLRP3	–	*In vivo*	(66, 67)
miR-155	SHIP-1	+	*In vivo* and *in vitro*	(48, 82)
miR-302b	IRAK4 EphA2	–	*In vivo* and *in vitro*	(68)
miR-488-3p	IL1-β	–	*In vitro*	(80)
miR-920	IL1-β	–	*In vitro*	(80)

a “-” indicates an inhibitory role during disease progression.

b “+” indicates a promoting role during disease progression.

### 3.3 The regulatory role of miRNAs in bone erosion

There is irreversible joint damage and deformity in advanced gout, mainly due to local cartilage damage and bone erosion caused by tophi (40). The primary mechanism is that MSU disrupts the balance between osteoblasts for bone formation and osteoclasts for bone resorption, decreases the activity of osteoblasts, promotes the aggregation and differentiation of osteoclasts, and promotes the development of inflammation and bone damage (84–86). Extensive studies have confirmed the involvement of miRNAs in the development of bone erosion. For instance, miR-20a targets RANKL through the TLR4/p38 pathway, hindering osteoclast proliferation and differentiation (87). Sujitha, S et al. found that miR-23a altered the expression level of LRP5 through RNA interference and contributed to a decrease in bone loss and an increase in calcium retention (88). In addition, Najm, A. et al. also demonstrated that miR-17 inhibits the autocrine effects of the IL-6 family *in vivo* by directly targeting JAK1 and STAT3 to exert anti-inflammatory and anti-bone erosion effects (89). There are few studies on miRNAs affecting bone erosion in gouty arthritis. Only An L et al. reported that miR-192-5p could inhibit MSU-induced EREG expression in GA mice to alleviate bone erosion (79). This case suggests that the underlying molecular mechanisms of miRNAs affecting bone erosion in GA remain to be explored.

## 4 Application of miRNAs in clinical diagnosis and treatment of GA

First of all, miRNA widely exists in a variety of body fluids, such as whole human blood (90), urine (91) saliva (92). Secondly, miRNA is stable in body fluid in a specific secretion mode, and it is easy to extract tissue samples without invasion. Even under changing environmental conditions, miRNA can stably exist. Because of its specificity, sensitivity, and stable expression in a wide range of diseases, MiRNA has early diagnostic capabilities and is rapid and accurate (92). In studies of gouty arthritis, several miRNAs are up-or down-regulated, and some of these miRNAs also vary with the extent of the disease. BohatáJ et al. found elevated levels of five circulating miRNAs, miR-17, miR-18a, miR-30c, miR-142, and miR-223, in the plasma of patients with GA and HUA (93). In addition, it has been reported that miR-221 is lowly expressed in the serum of AGA patients and the receiver operating curve (ROC) applied to the diagnostic value analysis showed an area under the curve of 0.884 (81). Therefore miRNAs have the potential to become markers for gout diagnosis.

The treatment of GA is mainly divided into anti-inflammatory and analgesic, and uric acid lowering. The primary treatment for gout attacks is cortisol, non-steroidal anti-inflammatory drugs (including selective and non-selective COX2 inhibitors), and low-dose colchicine to control pain and lessen inflammation (94–97). Although IL-1 inhibitors can effectively control gout attacks, they are usually reserved for patients with intolerable side effects or contraindications to first-line anti-inflammatory therapy (98, 99). The first-line uric acid-lowering therapy drug is allopurinol, a xanthine oxidase inhibitor. Still, patients who do not respond to or are intolerant of allopurinol are treated with febuxostat (6, 100). Probenecid, sulfinpyrazone, and benzbromarone can be used as monotherapy or combined with xanthine oxidase inhibitors by promoting uric acid excretion (101, 102). Liu, P et al. found that colchicine upregulated mir-223-3p and downregulated IL-1β, and etoricoxib treated AGA by upregulating miR-451a and downregulating COX-2 (103). A recent study reported that two novel hexapeptides (GPAGPR and GPSGRP) found in Apostichopus japonicus hydrolysates inhibit uric acid biosynthesis and reabsorption. The expression profiles of GPSGRP-treated HUA model mice were analyzed, and 21 differentially expressed miRNAs were identified (104).

Chinese medicine or natural products have been developed to treat gout arthritis in recent years. The drugs for acute gout arthritis have severe adverse reactions such as bone marrow suppression, liver cell damage, and gastrointestinal bleeding (105). Traditional Chinese medicine has some advantages in terms of low toxicity and adverse reactions. Wang Y et al. indicated that both Chuan Hu Tong Feng Compound and Allopurinol upregulated miR-486-5p, miR-339-5p, and miR-361-5p expression and decreased CCL2 and CXCL8 protein levels in patients with chronic gouty arthritis (106). Another research proved that benzbromarone and Xiezhuo Chubi Decoction reduced uric acid levels by increasing the expression levels of miR-34a and miR-146a (107). In addition, like Tripterine (77), Total glucosides of paeony (78) and Epigallocatechin (57) were also reported to regulate the expression of miR-449a, miR-876, and miR-9, respectively, to alleviate the progression of GA. Similarly, Li, X et al. reported that Noni (Morinda citrifolia L.) fruit Juice also modulates miRNA and pro-inflammatory factors to treat MSU-induced AGA in mice (108).

Although miRNA has much fundamental research on the treatment of arthritis, there are still many problems in the transition from mechanism research to clinical application. Therefore, miRNAs related to gout treatment need further exploration and development.

## 5 Future expectation

Since the discovery of miRNAs, their wide range of biological effects have been gradually revealed, also indicating that they play an important regulatory role in various cellular activities. Based on recent research results in related fields, in this section, we will look at the future directions of miRNA research and the prospects of clinical applications.

Exosomes, a cellular vesicle structure widely found in body fluids, have been identified for the presence of miRNAs. In recent years, several studies have demonstrated that miRNAs regulate inflammatory immunity and tumor progression through exosomes as vectors. Jiang, K et al. found that peripheral-derived exosome-mediated miR-155 promoted the polarization and proliferation of macrophage M1 and activated the NF-KB pathway to promote the release of inflammatory factors TNF-α and IL-6 in an acute lung injury model in mice (109). Similarly, Yingying Cao et al. reported that Enterotoxigenic Bacteroides fragilis (ETBF) promotes intestinal inflammation and malignancy by inhibiting exosome-encapsulated miR-149 (110). Naïve bone marrow-derived macrophages produce exosomes with anti-inflammatory miRNAs that target receptor macrophages to promote their M2 polarization and alleviate inflammation (111). Therefore, it is natural to speculate that circulating exosomes contain miRNAs that may influence the development of GA by regulating macrophage polarization or other key molecules of inflammation. Furthermore, such exosome-derived miRNAs have great application in both the diagnosis and treatment of GA.

In addition, nanomaterials as carriers of drug-targeted delivery systems have become a hot research topic due to the development of the interdisciplinary intersection of materials science and medicine in recent years. Since miRNA mimics are not resistant to nucleic acid endonucleases and are prone to degradation in circulation, nanomaterials can be used to wrap miRNAs for targeted therapy. Wang, F et al. reported that microRNA-31 bound to adriamycin-loaded mesoporous silica nanoparticles would be used to target tumor cells high in MTEF4 expression to promote mitochondrial apoptosis (112). Moreover, Ahir, M. et al. also reported that the use of mesoporous silica nanoparticles as co-delivery carriers of miR-34a-Mimic and antisense-miR-10b on tumor cells effectively inhibited tumor growth and metastasis in triple-negative breast cancer (113). Although nanomaterials are being studied as potential therapies for tumors and inflammatory diseases, they may still cause an immune response in the body and exacerbate inflammation. Therefore the development of low inflammatory response nanomaterials for the treatment of inflammatory diseases remains a great challenge. With the development of basic science and technology, gene-editing technology is becoming more and more mature and is expected to be used for the treatment of many diseases. CRISPR/Cas9 technology, which won the Nobel Prize, has brought a revolution to the life science field. Recently, Zhou, W et al. reported that CRISPR/Cas9-mediated knockdown of miRNA-363 effectively promoted apoptosis in diffuse large B-cell lymphoma cell lines in response to adriamycin-induced apoptosis (114). And, Yu Toyoda et al. also utilized CRISPR/cas9 to construct knockout mice to identify the role of GLUT12 in regulating blood urate levels (115). Therefore, the use of CRISPR/cas9-mediated miRNA knockdown to suppress inflammation in gouty arthritis still holds great promise for research.

## 6 Conclusion

In recent years, miRNAs have become a hot topic in biomedical research. Current studies have shown that miRNAs are closely associated with the development and progression of gouty arthritis, and miRNAs play an essential role in the post-transcriptional regulation process of genes. Despite the large number of studies reporting miRNA regulation of the gouty inflammatory process, there is still a significant gap in gouty arthritis, especially in bone erosion.

The underlying mechanisms of self-remission of gout as a recurrent chronic disease and the formation of gouty stones remain unclear. What is neutrophil-associated NETosis in the inflammatory process, and is it the culprit that exacerbates inflammation or relieves it leading to recurrent gout attacks?

Although gout is already a treatable rheumatic disease, the side effects of drugs are still evident, and targeting miRNAs may provide a new idea and insight for gout treatment. In the future, miRNA is expected to be a marker for diagnosing gouty arthritis or a target for drug therapy. However, further studies are still needed. Therefore, the search for relevant miRNAs and further study of their mechanisms are essential for diagnosing and treating gouty arthritis.

## Author contributions

TX and JWu conceived and designed the article, ZL and FY wrote the manuscript, SH and JWa reviewed the literature, BC, LL and JY revised the article, and YY, CY, YH, and SW proofread the language. All authors contributed to the article and approved the submitted version.

## Funding

This study was supported by the youth scientific research fund of Anhui Medical University (2021xkj275, chaired by zhipan Luo) and the school level quality project of Anhui Medical University (2021xjyxm24, chaired by zhipan Luo).

## Conflict of interest

The authors declare that the research was conducted in the absence of any commercial or financial relationships that could be construed as a potential conflict of interest.

## Publisher’s note

All claims expressed in this article are solely those of the authors and do not necessarily represent those of their affiliated organizations, or those of the publisher, the editors and the reviewers. Any product that may be evaluated in this article, or claim that may be made by its manufacturer, is not guaranteed or endorsed by the publisher.
